# Effects of *APOE4* allelic dosage on lipidomic signatures in the entorhinal cortex of aged mice

**DOI:** 10.1038/s41398-022-01881-6

**Published:** 2022-03-29

**Authors:** André Miguel Miranda, Archana Ashok, Robin Barry Chan, Bowen Zhou, Yimeng Xu, Laura Beth McIntire, Estela Area-Gomez, Gilbert Di Paolo, Karen E. Duff, Tiago Gil Oliveira, Tal Nuriel

**Affiliations:** 1 Life and Health Sciences Research Institute (ICVS), School of Medicine, University of Minho, Campus Gualtar, 4710-057 Braga, Portugal; 2 ICVS/3B’s - PT Government Associate Laboratory, Braga/Guimarães, Portugal; 3 Neuroradiology Unit, Department of Imagiology, Centro Hospitalar Vila Nova Gaia/Espinho, 4434-502 Vila Nova Gaia, Portugal; 4 Taub Institute for Research on Alzheimer’s Disease and the Aging Brain, Columbia University, 630 West 168th Street, New York, NY 10032 USA; 5 Department of Pathology and Cell Biology, Columbia University, 630 West 168th Street, New York, NY 10032 USA; 6 Department of Neurology, Columbia University, 630 West 168th Street, New York, NY 10032 USA; 7 UK Dementia Research Institute, University College London, Cruciform Building, Gower Street, London, WC1E 6BT UK; 8 Department of Neuroradiology, Hospital de Braga, 4710-243 Braga, Portugal; 9 Present Address: Denali Therapeutics Inc., South San Francisco, CA 94080 USA

**Keywords:** Molecular neuroscience, Diseases

## Abstract

Apolipoprotein E ε4 (*APOE4*) is the primary genetic risk factor for the late-onset form of Alzheimer’s disease (AD). Although the reason for this association is not completely understood, researchers have uncovered numerous effects of *APOE4* expression on AD-relevant brain processes, including amyloid beta (Aβ) accumulation, lipid metabolism, endosomal-lysosomal trafficking, and bioenergetics. In this study, we aimed to determine the effect of *APOE4* allelic dosage on regional brain lipid composition in aged mice, as well as in cultured neurons. We performed a targeted lipidomic analysis on an AD-vulnerable brain region (entorhinal cortex; EC) and an AD-resistant brain region (primary visual cortex; PVC) from 14–15 month-old *APOE3/3*, *APOE3/4*, and *APOE4/4* targeted replacement mice, as well as on neurons cultured with conditioned media from *APOE3/3* or *APOE4/4* astrocytes. Our results reveal that the EC possesses increased susceptibility to *APOE4*-associated lipid alterations compared to the PVC. In the EC, *APOE4* expression showed a dominant effect in decreasing diacylglycerol (DAG) levels, and a semi-dominant, additive effect in the upregulation of multiple ceramide, glycosylated sphingolipid, and bis(monoacylglycerol)phosphate (BMP) species, lipids known to accumulate as a result of endosomal-lysosomal dysfunction. Neurons treated with conditioned media from *APOE4/4* vs. *APOE3/3* astrocytes showed similar alterations of DAG and BMP species to those observed in the mouse EC. Our results suggest that *APOE4* expression differentially modulates regional neuronal lipid signatures, which may underlie the increased susceptibility of EC-localized neurons to AD pathology.

## Introduction

Possession of the ε4 allele of apolipoprotein E (*APOE*) is the primary genetic risk factor for late-onset Alzheimer’s disease (AD). While early work investigating the association between *APOE* ε4 (*APOE4*) and AD focused on the ability of *APOE4* to increase the aggregation and decrease the clearance of amyloid beta (Aβ) [1–7], more recent work has uncovered numerous effects of differential *APOE* isoform expression on other AD-relevant systems in the brain, including intracellular trafficking, energy metabolism, inflammation, and vascular integrity [see reviews by [8–11]]. It is important to note, however, that the primary role of the apoE protein, in both the brain and the periphery, is to mediate the transport of cholesterol and other lipids [12–14], a function that is also significantly altered by *APOE4* expression [15–19]. Importantly, AD pathology and neuronal aging are linked to dysregulation of lipid metabolism [20], and specific lipid signatures provide diagnostic value in predicting phenoconversion to either amnestic mild cognitive impairment (aMCI) or AD [21]. Moreover, mitigation of cholesterol imbalances has been increasingly explored as a therapeutic strategy, with success in pre-clinical models of AD [22, 23].

While several groups have addressed *APOE* isoform-related lipid alterations in plasma and serum [24, 25], relatively few studies have reported the use of lipidomic analysis to investigate the lipid alterations that occur specifically in the brain [26–31]. The results from these studies include observations that *APOE4* expression increased the levels of triacylglycerol (TAG) and decreased the levels of lysophosphatidylcholine (LPC), phosphatidylserine (PS), cholesterol, and 14-demethylanosterol (a cholesterol derivative) in the brains of *APOE* targeted replacement mice [28, 30]. In addition, *APOE4* mice were more sensitive to lipid alterations caused by a high-fat and/or high-cholesterol diet, most notably showing increased levels of cholesterol-esters in the brain [28]. In humans, a post-mortem lipidomic analysis of the inferior parietal lobule from AD patients showed that *APOE4* carriers had decreased levels of glycerophospholipids, most notably in phosphatidic acid (PA) [29].

Although this data is informative, there is still much to learn about the effects of *APOE* genotype on lipid signatures in the brain, especially in regards to the region- and cell-type-specific effects of *APOE4* expression on individual lipid species. We have previously reported a multi-omic analysis on an AD-vulnerable brain region (entorhinal cortex; EC) and an AD-resistant brain region (primary visual cortex; PVC) of aged *APOE* targeted replacement mice, which has thus far revealed novel effects of *APOE4* expression on several important biological pathways, including neuronal activity [32], endosomal-lysosomal trafficking [33], and bioenergetics [34]. Here, we present the results from our lipidomic analysis, which reveals specific effects of *APOE4* expression on the regional lipid signatures of the EC and PVC from aged *APOE* mice, as well as results from a complementary lipidomic analysis on mouse primary neurons treated with conditioned media from *APOE4/4* vs. *APOE3/3* expressing astrocytes. Our findings reveal region- and cell-type-specific *APOE4*-associated lipid alterations in the brain, thus shedding light on novel features relevant for AD pathogenesis independent of Aβ.

## Materials and methods

### Mice

Human *APOE* targeted replacement mice were first developed by Sullivan et al. [35, 36] and were acquired from Taconic Biosciences or directly from Dr. Patrick Sullivan. All mice used in this study were treated in accordance with the National Institutes of Health Guide for the Care and Use of Laboratory Animals and approved by the Columbia University Irving Medical Center Institutional Animal Care and Use Committee (IACUC). For this study, we utilized 14–15 month-old male *APOE3/3*, *APOE3/4*, and *APOE4/4* mice, 8 mice per genotype. This sample size was chosen based on similar lipidomics studies in mice [37, 38], and our own metabolomics study in *APOE* mice [32].

### Extraction of lipids from brain tissue

Mice were sacrificed by cervical dislocation to maintain the brain environment, and individual brain regions were immediately removed and snap-frozen on dry ice. Tissues were stored at −80 °C prior to extraction. Lipid and small-molecule metabolite extraction was performed using a methyl tert-butyl ether (MTBE)/methanol extraction protocol modified from previous reports [39, 40], as we have described previously [32]. Briefly, individual EC or PVC tissues were homogenized in 400 μl of ice-cold methanol using a bead mill homogenizer (TissueLyser II, Qiagen) at 25 beats/sec, 2x for 45 sec each. Following homogenization, samples were incubated in 1200 μl of MTBE for 1 hr at room temperature to separate organic-soluble lipids from aqueous-soluble lipids and other small-molecules. Finally, 360 μl of ultrapure water was added (for a final ratio of 3:1:0.9 MTBE:methanol:water) to resolve the two liquid phases, and each samples were centrifuged at 10,000 x *g* for 10 min. For this experiment, the upper organic phase was collected from each sample and stored in a separate tube, and the remaining protein pellets were resuspended in 25 mM ammonium bicarbonate, pH 8, with 2.5% SDS. A BCA protein assay was performed on each protein fraction, and the organic phase was normalized to their protein concentration equivalent with 100% methanol. All samples were then stored at −80 °C prior to analysis.

### Extraction of lipids from astrocyte conditioned media-treated primary neurons

Astrocyte conditioned media (ACM) was obtained from immortalized astrocyte cell lines (a gift from Dr. David Holtzman) that were originally generated from primary astrocytes from P1-2 pups of *APOE* targeted replacement mice [41]. The immortalized astrocytes were conditioned with Neurobasal media supplemented with B27, Glutamax-I, Normocin and 1% penicillin/streptomycin for 24 h. This ACM was then collected, stored at −80 °C, and thawed prior to use. WT primary cortical neuronal cultures were obtained from embryonic day 17 (E17) C57Bl/6 (B6) embryos. Briefly, pregnant mice were euthanized by cervical dislocation and the embryo brains extracted. The meninges were removed and the cortices dissected out. The cortices were then enzymatically dissociated in 0.25% Trypsin-EDTA and resuspended in Neurobasal media supplemented with B27, Glutamax-I, Normocin and 1% penicillin/streptomycin. Dissociated cells were counted and 300 K neurons per well were plated directly into ACM in poly-D-lysine (PDL)-coated 6-well plates. ACM-treated neurons were then incubated at 37 °C for 7 days in a humidified chamber with 5% CO_2,_ with 50% media exchange (with newly thawed ACM) once every 3 days. The neurons were then harvested, and the lipids were extracted using the MTBE/methanol extraction protocol described above. All samples were then stored at −80 °C prior to analysis.

### Lipidomic analysis

Lipid profiling was performed using an Agilent 1260 HPLC coupled to an Agilent 6490 triple quadrupole (QQQ) mass spectrometer [42]. Each sample was run through three separate chromatographic conditions (reverse-phase negative mode, reverse-phase positive mode and normal-phase positive mode) for the effective quantification of 337 distinct lipids from 28 lipid subclasses, as previously described [42]. From the mouse analysis, four samples (two *APOE3/4* EC samples, one *APOE3/3* PVC sample, and one *APOE4/4* PVC sample) were removed prior to data processing due to chromatographic errors during the runs. No samples were removed prior to data processing from the cell analysis. Individual lipid species were measured by multiple reaction monitoring transitions and lipid concentration was calculated by referencing appropriate internal standards per lipid class added to each sample, using Agilent’s MassHunter Quantitative Analysis software (version 5). Lipid classes not internally standardized owing to commercial unavailability of respective standards were referenced to closely eluted standards. In mice, relative free cholesterol levels were calculated as relative mol% of all lipids detected for each sample; all other lipid species are expressed as relative mol% of all lipids measured except free cholesterol. In cultured neurons, all lipids are expressed as relative mol% of all lipids measured [43]. Blinding was performed during both the data acquisition and the data processing steps. Lipid nomenclature follows LIPID MAPS consortium guidelines. Lipid species are annotated as lipid class followed by total number of carbons and unsaturation degree of acyl chains. DAG and TAG species are annotated with the addition of acyl carbon and unsaturation of the product ion (e.g., DAG30:0/14:0). Sphingolipids contained d18:1 long-chain base except dhSM species, containing a d18:0 base. N-acyl-PS species contained C16:0 N-linked acyl chains.

### Statistics

Statistical analysis was performed using SPSS Statistics 26 (IBM) and Prism 8.0 (Graphpad) software. Animals were randomly recruited to experimental groups based on genotype. Sample size was based on previous studies [44, 45], and is indicated in each legend. Samples were blinded during tandem biochemical analysis. Normality and homogeneity of variance were assessed with Shapiro–Wilk and Levene’s test, respectively. One-way ANOVA followed by Tukey’s post-hoc was performed for multiple comparisons and Kruskal–Wallis test was used for non-parametric testing, as referenced in each figure legend. A confidence interval of 95% was assumed for all statistical tests. No samples or animals were excluded from analysis.

## Results

### Regional effects of *APOE4* expression on brain lipid composition

In order to understand the effects of differential *APOE* isoform expression on lipid signatures in the brain, we performed a targeted analysis of lipid metabolites extracted from the brains of aged *APOE* mice. Specifically, we utilized male 14–15 month-old *APOE* targeted replacement mice, which express the human *APOE* gene in place of the mouse *Apoe* gene. In order to understand the regional and dosage effects of *APOE4* allelic expression, we studied *APOE3/3*, *APOE3/4*, and *APOE4/4* mice (*n* = 8 mice per group) and extracted lipids from two brain regions: the EC, which is one of the first brain regions where neurofibrillary tangles (NFTs) accumulate in AD, and the PVC, which develops tangle pathology at a later stage [46]. The lipidomic analysis was performed using a previously described liquid chromatography-mass spectrometry (LC-MS) platform [42], allowing for the detection of 28 lipid classes and over 300 distinct lipid species (Extended Data File).

**Fig. 1:**
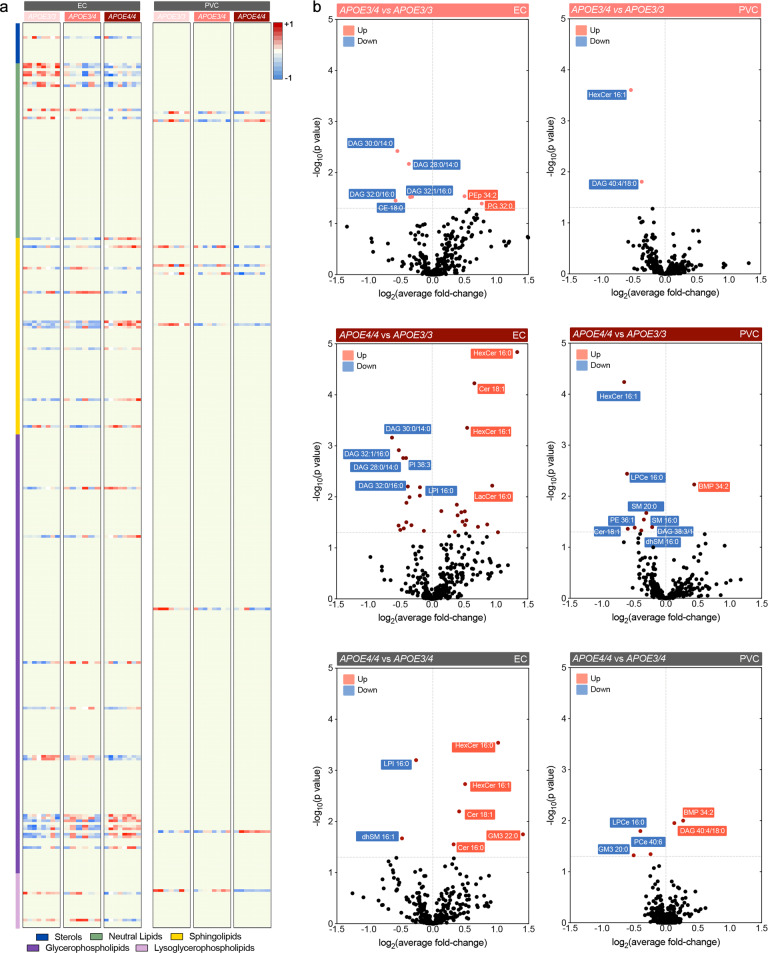
Comparative lipid profile of EC and PVC of aged *APOE* targeted replacement mice. **a** Heatmap of lipids significantly altered by *APOE3/4*, *APOE4/4* and *APOE3/3* genotype in EC and PVC at 14–15 months of age. Each row represents a lipid species of a given category (see color legends); each column represents a sample. Results are expressed as Z-score [(average mol% of lipid species per genotype−average mol% of lipid species in all genotypes)/standard deviation of average mol% lipid species] represented in gradient color; blue and red indicate negative and positive Z-score, respectively. One-way ANOVA was performed and results thresholded by *p* < 0.05; *n* = 8, 6, and 8 for EC and *n* = 7, 8, and 7 for PVC in *APOE3/3*, *APOE3/4*, and *APOE4/4* mice, respectively. **b**, **c** Volcano plots of differentially modulated lipid species in EC and PVC between *APOE3/4* vs. *APOE3*^*/*^*3*, *APOE4/4* vs. *APOE3*^*/*^*3* and *APOE4*^*/*^*4* vs. *APOE3*^*/*^*4*. Colored plots denote significantly affected species at Tukey’s post-hoc *p* < 0.05, black plots denote unaltered species (*p* > 0.05).

Our results confirm that cholesterol is the most abundant lipid subclass in the brain, followed by phosphatidylcholine (PC), phosphatidylethanolamine (PE)/plasmalogens, and sphingomyelin (SM), all of which play structural roles in the assembly of cellular membranes [47] (Extended Data File). While our findings corroborate previous observations reporting that differential *APOE* isoform expression does not profoundly alter lipid composition in the brain [26, 27], significant effects were observed in the levels of less abundant and bioactive lipid species in both brain regions. In the EC, 35 lipid species were differentially expressed in aged *APOE* mice, whereas only 9 lipid species were differentially expressed in the PVC between the different genotype groups (Fig. 1a).

Specifically, we observe that in the EC, several neutral lipid species were affected by *APOE4* expression (Fig. 1a), including numerous short-length diacylglycerol species (DAG 28-34 C), which showed decreasing levels with *APOE4* expression, as did several longer DAG species (38-40 C). Cholesterol-ester (CE) 18:0 was also reduced in the EC with increasing number of *APOE4* alleles. We detected differential effects in sphingolipid species, namely *APOE4*-associated increases in ceramides (Cer) 16:0 and 18:1; hexosylceramides (HexCer) 16:0, 16:1, 18:0, and 26:0; lactosylceramide (LacCer) 16:0; and monosialodihexosylganglioside (GM3) 22:0, while sphingomyelin (SM) 18:0 and dihydrosphingomyelin (dhSM) 16:1 were decreased with *APOE4* expression. For glycerophospholipids, phosphatidylcholines (PC) 36:0 and 42:7; plasmalogen phosphatidylethanolamine (PEp) 34:2; and PS 42:4 and 42:5 were increased with *APOE4* expression, while phosphatidylglycerol (PG) 32:0 and phosphatidylinositols (PI) 38:3 and 38:4 were decreased. In addition, lysoglycerophospholipids, such as lysophosphatidylinositol (LPI) 16:0 and lysoetherphosphatidylcholine (LPCe) 18:0, were also decreased. Interestingly, multiple species (32:0, 34:0, 34:1, 36:0, 36:1, and 38:4) of the atypical phospholipid bis(monoacylglycerol)phosphate (BMP) [which is also commonly referred to as lysobisphosphatidic acid (LBPA)] were elevated with increasing *APOE4* alleles. Importantly, while BMP is exclusively found in the intraluminal vesicles (ILVs) of late endocytic compartments and is considered a bona fide marker of these structures [48], we have also previously observed increased levels of BMP in the EC of late-onset AD patients [42].

In the PVC, we found decreased levels of DAG 38:3, Cer 18:1, SM 16:0, SM 20:0, PE 36:1, and LPCe 16:0 with *APOE4* expression, while BMP 34:2 was significantly increased (Fig. 1a, right panel). These observations support a reduced effect of *APOE4* on the lipid composition of the PVC as compared to the EC, which may be due to the fact that *APOE* displays increased gene expression in the medial temporal lobe compared to other brain regions [49, 50]. We also note that most of the differentially expressed lipid subclasses we observed are affected in both regions, namely DAG, Cer, SM, and BMP, albeit to a lesser degree in the PVC. Remarkably, these lipids are all associated with the endosomal-lysosomal pathway, as either bioactive signaling molecules (DAG) [51], substrates of lysosomal degradation (Cer, SM) [52], or lysosomal resident lipids (BMP) [48]. This suggests that while some effects of *APOE4* expression may be regionally restricted, others are common throughout the brain, including previously described effects of *APOE4* expression on endosomal-lysosomal trafficking [33, 53].

### Co-dominant and additive effects of *APOE4* alleles in the EC

In terms of AD susceptibility, one copy of *APOE4* confers a ~3-fold increased risk, while two copies of *APOE4* confers a ~12-fold increased risk of developing AD [14]. For this reason, it is important to understand which biological effects of *APOE4* expression are in agreement with this additive model (i.e., a second *APOE4* allele has an additional impact), and which features are associated with a dominant *APOE4* effect (i.e., it is common to either one or two alleles of *APOE4*). To better understand the effect magnitude of each *APOE4* allele on lipid signatures, we performed paired comparisons between the three *APOE* genotypes (*APOE3/3*, *APOE3/4*, and *APOE4/4;* Fig. 1b, c). Using post-hoc multiple comparison analysis, we detected 7 and 31 significantly altered lipid species in the EC when *APOE3/4* or *APOE4/4* mice, respectively, were compared to *APOE3/3* mice (Fig. 1b). Notably, 71% (5 of 7) of the lipid species altered in the *APOE3/4* vs. *APOE3/3* EC were also significantly affected in the *APOE4/4* vs. APOE3/3 EC. In the PVC, we detected 2 and 9 significantly altered species in *APOE3/4* or *APOE4/4* mice, respectively (Fig. 1c).

**Fig. 2:**
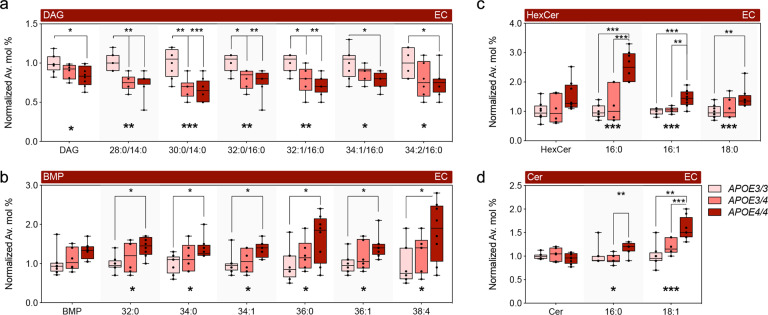
Dominant and semi-dominant effects of *APOE*_4_ expression on lipid composition in the EC in aged *APOE* mice. **a**–**c** Normalized average mol% (fold-change) of (**a**) DAG, (**b**) BMP, (**c**) Hex and (**d**) Cer lipid species from the EC of aged *APOE* targeted replacement mice. Lipids were annotated per total acyl carbons and degree of unsaturation. Data represent median ± max/min. One-way ANOVA followed by Tukey’s post-hoc test for multiple comparisons was performed; *n* = 8, 6, and 8 for EC and *n* = 7, 8, and 7 for PVC in *APOE3/3*, *APOE3/4*, and *APOE4/4* mice, respectively. Kruskal–Wallis H test confirmed statistically significant difference in levels of BPM 32:0 (*p* = 0.03), HexCer 16:0 (*p* = 0.003) and Hex Cer 16:1 (*p* = 0.003). *p < 0.05, **p < 0.01, and ***p < 0.001.

Of the significantly altered species in the EC, DAG 28:0, 30:0, 32:0, and 32:1 were decreased in *APOE3/4* and also ranked within the top-10 most significantly altered in *APOE4/4* mice (Fig. 1b and 2a). Since no significant differences were detected in these species from the *APOE4/4* vs. *APOE3/4* EC paired comparison, these findings suggest a dominant effect of *APOE4* on DAG levels (Fig. 2a). Box-plot representations of the average fold-change of lipid levels across each of the three genotypes confirm the dominant effect of *APOE4* in DAG levels (Fig. 2a). Additionally, we found a two-step additive effect in specific BMP species (Fig. 2b), as well as Cer 16:0, 18:1, and more complex glycosphingolipids, such as HexCer 16:0, 16:1, 18:0 (Fig. 2c, d), and GM3 22:0, in which significant changes were only detected in the *APOE4/4* vs. *APOE3/3* EC. The only decreased species in *APOE4/4* vs. *APOE3/4* EC were dhSM 16:1 and LPI 16:0. Altogether, our findings indicate a dominant effect of *APOE4* on DAG levels, with a semi-dominant and additive effect on Cer, glycosylated sphingolipids, and BMP.

### Acyl chain profile is not affected by *APOE*_*4*_ genotype in the EC and PVC

The chemical structures of lipids are a determinant of their physical/chemical properties, which in turn determines the biological roles that they play in cells and tissues. While lipid categorization is largely determined by its polar head group, its acyl chain structure further contributes to membrane compositional diversity, which differs greatly between tissues and subcellular compartments [47]. Importantly, lipid-metabolizing enzymes and transporters present preferred affinity to certain acyl chains; as such, acyl chain profiles may indicate selective modulation of specific metabolic pathways. For these reasons, we analyzed the effect of *APOE4* alleles on the acyl chain profile of DAG/glycerophospholipids (GPL) and sphingolipids (SL) in the EC and PVC.

Despite the dominant effect of *APOE4* on decreasing the levels of short-length DAG species in the EC, no global impact was observed in the acyl chain length for DAG/GPL or for SL in *APOE3/4* or *APOE4/4* mice, as compared to *APOE3/3* mice (Supplementary Fig. 1a, b). Similarly, no overall impact was observed on the degree of saturation of either lipid category. While we have previously reported an effect at the level of free fatty acids in the EC of *APOE4* mice, including an increase in docosahexaenoic acid (22:6) in the EC [34], our results do not support a preferential acyl chain enrichment in the molecular signature of DAG/GPL and SL.

### Effects of *APOE* astrocyte conditioned media on neuronal lipid composition

A present limitation of performing whole tissue lipidomic analysis is the inability to discern the corresponding contribution of each cell-type present within that tissue. To overcome this constraint, we used the paradigm of in vitro conditioned media transfer to analyze the effect of humanized *APOE4/4* vs. *APOE3/3* expression in astrocytes (the primary source of apoE protein in the brain) on the lipid composition of wild-type, B6 cortical neurons. Specifically, ACM was collected from immortalized astrocytes generated from post-natal day 1-2 (P1-2) pups of *APOE3/3* or *APOE4/4* targeted replacement mice [41]. Primary neurons derived from embryonic day 17 (E17) B6 mice were plated in either no ACM, *APOE3/3* ACM, or *APOE4/4* ACM (*n* = 6 wells per group) and incubated for 7 days, before harvesting for lipidomic analysis.

In comparison to untreated cells, the ACM dramatically altered neuronal lipid composition, with overlapping effects regardless of *APOE* isoform (Supplementary Fig. 2a and Extended Data File). We observed a 2-fold increase in DAG levels and a more modest elevation of SL levels, including SM, dhSM, HexCer, and LacCer (Supplementary Fig. 2b). For GPL, PA, and phosphatidylglycerol (PG) levels were decreased, whereas the products of PG transformation, acyl-PG and BMP, were increased by 1.3-fold and a dramatic 8-fold average, respectively (Supplementary Fig. 1c). Also, ether phosphatidylcholines (PCe) and LPCe were increased by *APOE* ACM, although to a different extent by each isoform. Thus, ACM alone induces significant remodeling of neuronal membranes, a fundamental characteristic to account for in future co-culture experiments.

**Fig. 3:**
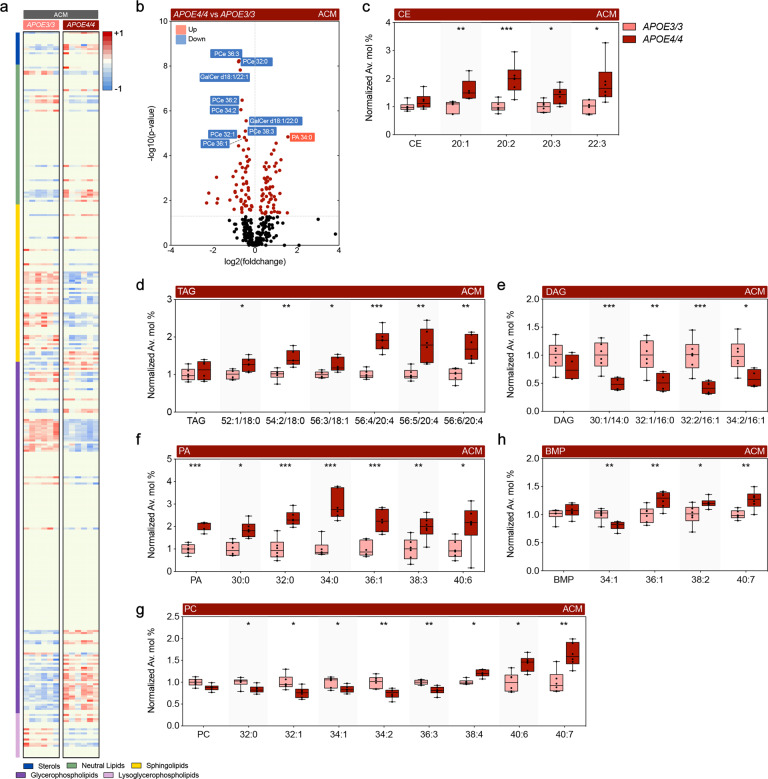
Comparative effect of *APOE*_*3/3*_ versus *APOE*_*4/4*_ astrocyte conditioned media (ACM) on the lipid profile of wild-type (WT) neurons. **a** Heatmap of lipids significantly altered in WT neurons after treatment with ACM derived from immortalized *APOE3/3* and *APOE4/4* astrocytes. Results expressed as Z-score [(average mol% of individual lipid species per genotype−average mol% of lipid in all genotypes)/standard deviation of average mol% lipid] represented in gradient color; blue and red indicate negative and positive Z-score, respectively. One-way ANOVA was performed and results threshold by *p* < 0.05; *n* = 6. **b** Volcano plots of differentially modulated lipid species under same conditions as in (**a**). Colored plots denote significant alterations (Tukey’s post-hoc *p* < 0.05), black plots denote unaltered species (*p* > 0.05). **c**–**h** Normalized average mol% (fold-change) of (**c**) CE, (**d**) TAG, (**e**) DAG, (**f**) PA (**g**) PC, (**h**) BMP lipid species from WT neurons incubated with ACM. Lipids were annotated per total acyl carbons and degree of unsaturation. Data represent median ± max/min. One-way ANOVA followed by Tukey’s post-hoc test for multiple comparisons was performed. *p < 0.05, **p < 0.01, and ***p < 0.001.

Next, we compared the differential effects of *APOE4/4* vs. *APOE3/3* ACM on neuronal lipid signatures (Fig. 3a, b). We opted for the use of homozygous astrocytes for each isoform to increase the sensitivity of any additive effects of *APOE4*. Importantly, we found a 30% increase in free cholesterol levels in *APOE4/4* vs. *APOE3/3* ACM-treated neurons. Several CE species (20:1, 20:2, 20:3, and 22:3) and TAG species (52:1/18:0, 54:2/18:0, 56:3/18:1, 56:4/20:4, 56:5/20:4, 56:6/20:4) were increased (Fig. 3c, d). On the other hand, short-length DAG species (30:1/14:0, 32:1/16:0, 32:2/16:1, and 34:2/16:1) were decreased in *APOE4/4* vs. *APOE3/3* ACM-treated neurons (Fig. 3e), confirming an inverse effect of *APOE4* expression on DAG levels comparatively to neutral lipids. Concerning SL, we found increased levels of Cer 18:1/16:0 and SM 18:1/16:1 in the presence of *APOE4*, while longer SM 18:1/22:1 levels were decreased, as were multiple species of more complex and glycosylated sphingolipids, including HexCer, Sulf, LacCer, and GM3.

**Fig. 4:**
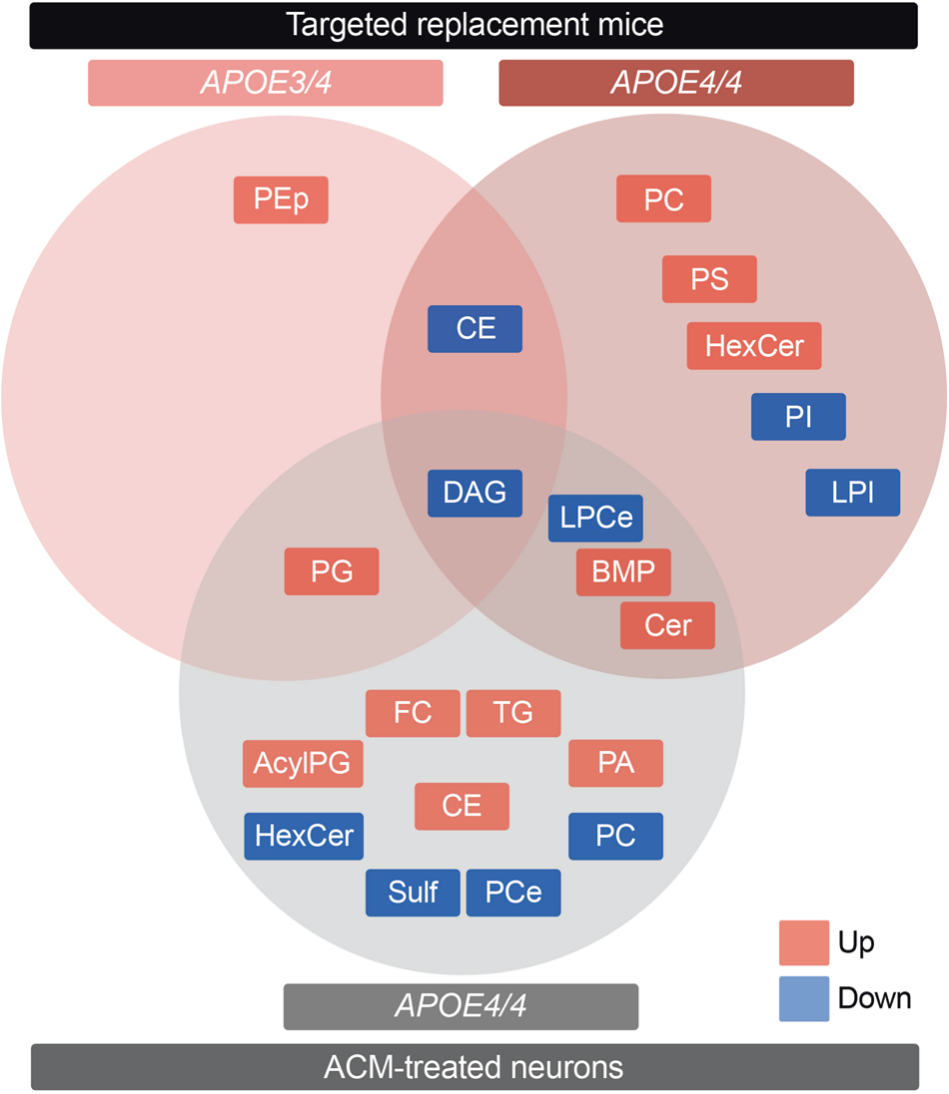
*APOE* isoform expression differentially modulates lipid profiles. Venn diagram generated from lipid species significantly altered in the EC in *APOE4/4* and *APOE3/4* targeted replacement mice *versus APOE3/3* mice (upper row) and in WT cortical neurons treated with *APOE4/4* versus *APOE3/3* ACM (lower row). Only lipid classes are represented; refer to Figs. 1–3 for specific lipid acyl-chain composition per class, respectively. Red denotes increased levels; blue denotes decreased levels.

GPL were also differentially modulated by the presence of humanized *APOE4/4* vs. *APOE3/3* ACM, including increases in multiple PA species (30:0, 32:0, 34:0, 36:1, 38:3, and 40:6) (Fig. 3f), whereas PC species (32:0, 32:1, 34:1, 34:2, and 36:3) were decreased (Fig. 3g), suggesting direct conversion of these metabolically linked lipids. Notably, longer PC species (38:4, 40:6, and 40:7) were increased in *APOE4/4* vs. *APOE3/3* ACM (Fig. 3g). We also observed a prominent decrease in several PCe species, spanning several degrees of acyl-chain length (30–38 C) and unsaturation (1-6 double bonds), while only PE 34:2, 36:2, and PEp 36:3 were decreased among ethanolamine-containing glycerophospholipids. Another synthetic pathway upregulated upon exposure to *APOE4/4* vs. *APOE3/3* ACM was PG (30:0, 32:1, 36:1, and 36:2), Acyl-PG (34:1, 34:2, 36:1, 36:2, 36:3, 36:4, 38:4, and 38:5), and BMP (36:1, 38:2, and 40:7), similar to what was observed in *APOE4/4* brains, while BMP 34:1 levels were decreased (Fig. 3h). Finally, LPC levels (18:0, 20:0 and 20:1) were increased, whereas LPCe (16:0 and 18:1) and LPI (16:0, 18:1, and 20:3) were decreased. Altogether, while we confirm that the previously observed effect of *APOE4* on DAG and BMP levels also occurs in ACM-treated neurons, we note an inverse effect of *APOE4* between PC and PCe levels and metabolically linked PA specifically in ACM-treated neurons (Fig. 4).

## Discussion

AD is a multi-faceted disease caused by both genetic and environmental factors. While hallmark AD pathology includes Aβ and tau accumulation, dysregulated lipid homeostasis has also been implicated in AD [42]. Furthermore, *APOE4*, the primary genetic risk factor for late-onset AD, is itself a regulator of lipid transport and metabolism. Our findings suggest that the EC, one of the first brain regions to develop AD pathology, presents increased susceptibility to *APOE4*-associated lipid alterations, as many more lipids were altered by *APOE4* expression in the EC than the PVC (Fig. 1). Interestingly, this corroborates findings from our previous transcriptomic analysis of *APOE3/4* vs. *APOE3/3* mice, where far more genes were differentially expressed by *APOE4* expression in the EC than in the PVC [33]. This differential effect may in part be related to the increased expression of *APOE* observed in the medial temporal lobe compared to other brain regions [49, 50]. Nonetheless, regional microstructure differences, such as cell-type distribution, fiber tract density or local enzyme expression, likely contribute as well.

Our data indicates that *APOE4* expression produces a dose-dependent accumulation of several lipids associated with endosomal-lysosomal trafficking, such as HexCer and BMP, in both the brain and in ACM-treated neurons (Figs. 1, 3, 4). Given that the number of *APOE4* alleles associates with increased risk for AD, we hypothesize that the additive effect on accumulation of lipids related to the endosomal-lysosomal system is a critical driver of prodromal neuronal pathology. In fact, these effects of *APOE4* are reminiscent of that observed in lysosomal storage disorders [54, 55]. Not only is accumulation of BMP a hallmark of Nieman-Pick type C (NPC), it is also observed in AD specimens and upon disruption of endolysosomal trafficking, reflecting expansion of late endosome volume and cholesterol storage [48, 56, 57]. Also, homozygous mutations in *GBA* and *GALC* impair clearance of HexCer, namely gluco- and galactosylceramides, and cause Gaucher’s and Krabbe’s disease, respectively. Notably, heterozygous GBA carriers present the largest genetic risk factor for developing idiopathic Parkinson’s disease (PD) [58]. Since *APOE4* also increases the risk for dementia in pure synucleinopathies and specifically exacerbates α-synuclein pathology [59, 60], we propose the disruption of lysosomal metabolism of glycosphingolipids as a common contributing factor in both forms of dementia. This association bears great therapeutical potential, as allosteric activators of glucocerebrosidase restore lysosomal function [61] and have recently been proven safe in humans [62], underscoring the possibility of extending their application to management of AD in *APOE4* carriers.

In keeping with a prominent effect of *APOE4* in endosomal-lysosomal trafficking, we previously showed increased expression levels of Rab GTPase genes, such as *Rab5b*, *Rab7* and *Rab9*, in the EC of aged *APOE4* mice [33], similar to that observed in cholinergic basal forebrain neurons in MCI and AD [63]. Importantly, Rab5 activation recapitulates prodromal features of AD, including endosomal enlargement and synaptic dysfunction [64], Rab7b granules are found in the EC of Lewy Body dementia patients [65], and altered Rab7 colocalizes with Aβ in apoE treated microglia [66]; that said, altered Rab GTPase levels are thus far correlative in *APOE4* background, and their individual contributions to pathology or therapeutic potential remain elusive [67]. Also, we previously reported increased expression of several V-type ATPase subunits [33], possibly in response to altered lysosomal pH and membrane composition [68, 69]. Since neurons upregulate the secretion of exosomes to mitigate lysosomal lipid and cargo burden [56], the inhibitory effect of *APOE4* expression in exosomes release [70] may also contribute to neuronal vulnerability. An association between endolysosomal defects and AD is additionally supported by recent genome wide association studies (GWAS), which identified novel loci related to endosomal-lysosomal trafficking, including *WDR81, SNX1, CSTB*, and *GRN* [71]. Thus, our lipidomic findings strengthen the association of *APOE4* to endosomal-lysosomal dysfunction in contributing to increased susceptibility to AD and other forms of dementia.

Another key finding we observed was a dominant effect of *APOE4* on DAG levels, which was recapitulated when exposing neurons to *APOE4* vs. *APOE3* ACM (Fig. 4). Interestingly, our previous transcriptomic analysis revealed decreased expression of *Lpin3*, *Plc1*, and *Plcg2* in the EC of *APOE3/4* vs. *APOE3/3* mice [33], which could potentially contribute to decreased DAG levels via decreased dephosphorylation of PA and decreased cleavage of PI. Concurrently, we observed an increase in multiple CE and TAG species upon *APOE4* ACM treatment, which may also implicate DAG consumption, as inhibition of DAG transferases rescues TAG burden in *APOE4* expressing astrocytes [72]. Interestingly, lipid droplet number and volume have previously been shown to increase in neurons treated with *APOE4* vs *APOE3* ACM as a result of both increased lipid droplet synthesis and decreased mobilization [73, 74]. Higher cholesterol levels in neurons also likely reflect decreased secretion, which instead accumulates in distinct subcellular compartments, such as lysosomes [75, 76]. As sequestration of excessive fatty acids into lipid droplets is critical to neuroprotection [77], decreased DAG availability may reduce the capacity to buffer metabolic challenges. As such, a high reliance of neurons on incorporation of fatty acids into lipid droplets could justify the dominant effect of *APOE4* on DAG levels versus other lipids. Furthermore, the previously observed decrease in the ability to mobilize lipid droplets in the presence of *APOE4* ACM [74], which the authors were not able to rescue by supplementation with recombinant apoE4 protein, likely reflects a loss-of-function effect against other *APOE* isoforms. This loss-of-function possibility is supported by the description of CE and BMP accumulation in the forebrain of *Apoe*^−/−^ mice [78], as well as increased HexCer levels in the corpus callosum (CC) upon *Apoe* ablation [79]. However, DAG levels are increased in the CC of *Apoe*^−/−^ mice, which may reflect regional specificity (e.g., enrichment for commissural tracts) or co-existing gain-of-function effects (e.g., downstream of neuronal receptor signaling) [80]. The milder effect of *APOE4* ACM on the accumulation of BMP, comparatively to *APOE4* mice, may result from the interaction with endogenous neuronal murine apoE within the endolysosomal compartments.

We also observed an effect of *APOE4* ACM on increasing PA and decreasing PC and PCe levels in the ACM-treated neurons (Fig. 3). PA is tightly linked to DAG metabolism via synthesis and catalysis by DAG kinase and lipin/PA phosphatase [81] and has been implicated in synaptic vesicle recycling and in neuronal plasticity mechanisms [82], which may underlie neuronal hyperactivity in aged *APOE4* mice [32]. Also, PC is converted to PA by phospholipase D1 (PLD1) or PLD2 isoenzymes [83], both of which have been implicated in AD [84] and shown to signal downstream of Aβ [85, 86] and *APOE* [87]. Since PC can similarly be converted to LPC through the action of phospholipase A2 (PLA2), the inverse relationship between PC and LPC levels upon exposure to *APOE4/4* ACM suggests upregulation of this pathway, also observed in several transgenic AD models [88, 89]. Interestingly, supplementation with choline, a precursor of PC, restores *APOE4*-induced lipid defects and highlights the therapeutical potential of dietary interventions targeting lipid metabolism [72]. Additionally, we observed a neuron-specific decrease in multiple PCe lipid species, which is also observed in the prefrontal cortex [45] and ventral hippocampus [44] of rodents exposed to chronic stress, a known risk factor for AD [90]. As PCe species are natural scavengers of reactive oxygen species [91], this alteration may result in decreased neuroprotection. Recently, Fitz et al. showed that *APOE* particles in ACM from *APOE4* targeted replacement mouse primary astrocytes presented overall decreased levels of most GPL classes compared to *APOE3* particles [92]. This suggests that the more complex GPL changes we observe in neurons are not simply due to differences in the amounts of lipids received from the ACM, but to lipid metabolism occurring in *APOE4* vs. *APOE3* ACM-treated neurons.

While our results complement previous studies on *APOE* biology and AD, we acknowledge the limitations of our lipidomic snapshot, taken from a single age in male *APOE* mice and a single *APOE* treatment condition in cultured WT neurons. We anticipate that additional complexity in *APOE4*-associated lipid signatures will likely be uncovered when investigating the effects of differing ages, sex, and brain regions in vivo, or additional cell-types or neuronal environments in vitro. Indeed, aging has been shown to be associated with blood brain barrier (BBB) breakdown in the hippocampus [93], and BBB leakage is also found to be an early marker of dysfunction in both *APOE4* carriers [94] and mice [95]. Whether the lipid regional changes induced by *APOE4* are a cause or a consequence of BBB breakdown is still an intriguing unanswered question. Further caution is also warranted due to the observation that lipidome complexity increases from mice to humans [96], and *APOE* lipid effects may diverge between mice and humans [76].

In summary, our findings demonstrate increased susceptibility of the EC to *APOE4*-associated lipid alterations and implicate endosomal-lysosomal lipid flux and lipid droplet regulation as potential factors in the increased risk of AD and other forms of dementia among *APOE4* carriers. These findings support a role for lipid dyshomeostasis in selective vulnerability to AD pathology and highlight the therapeutic potential of lipid modulation in neurodegeneration.

## Supplementary information

Summplementary Information

Extended Data File

## Data Availability

The full lipidomic datasets are included as an additional Extended Data File and can also be found on the Metabolomics Workbench data repository at 10.21228/M82690.
