# Supplementary material for: Effects of APOE4 allelic dosage on lipidomic signatures in the entorhinal cortex of aged mice
Source: Transl Psychiatry. 2022 Mar 29;12:129. doi: 10.1038/s41398-022-01881-6 (PMC8964762; doi:10.1038/s41398-022-01881-6)
Supplement: Supplementary file 1 — Summplementary Information [file 41398_2022_1881_MOESM1_ESM.docx]

**Supplementary Information**

**Effects of *APOE4* allelic dosage on lipidomic signatures in the entorhinal cortex of aged mice**

André Miguel Miranda^1,2,3^, Archana Ashok^4,5^, Robin Barry Chan^4,5^, Bowen Zhou^4,5^, Yimeng Xu^4,5^, Laura Beth McIntire^4,5^, Estela Area-Gomez^4,6^, Gilbert Di Paolo^4,5,7^, Karen E. Duff^4,5,8^, Tiago Gil Oliveira^1,2,9,*^, Tal Nuriel^4,5,*^

**FIGURE LEGENDS**

**Supplementary Figure 1. Effects of *APOE4* expression in lipid acyl chain composition.** Average mol% of acyl chain profile of glycerophospholipid (GPL)/DAG and sphingolipids (SL) from the **(a)** EC and **(b)** PVC of aged *APOE4* targeted replacement mice. Lipids were annotated per total acyl carbons (length) and degree of saturation. Data represent median ± max/min. One-way ANOVA followed by Tukey’s post-hoc test for multiple comparisons was performed; n = 8, 6, and 8 for EC and n= 7, 8, and 7 for PVC in *APOE3/3*, *APOE3/4* and *APOE4/4* mice, respectively. No statistically significant results were found (p>0.05).

**Supplementary Figure 2. Comparative effect of astrocyte conditioned media (ACM) on the lipid profile of wild-type (WT) neurons. a)** Heatmap of lipid subclasses significantly altered in WT neurons after treatment with ACM derived from immortalized *APOE3/3* and *APOE4/4* astrocytes compared to control media (NO ACM). Results expressed as Z-score [(average mol% of individual lipid species per treatment−average mol% of lipid of all samples)/standard deviation of average mol% lipid] represented in gradient color; blue and red indicate negative and positive Z-score, respectively. One-way ANOVA was performed and results threshold by p<0.05; n=6. b) Normalized average mol% (fold-change) of lipid subclasses from WT neurons incubated with ACM or NO ACM. Data represent median ± max/min. One-way ANOVA followed by Tukey’s post-hoc test for multiple comparisons was performed. *p < 0.05, **p < 0.01, and ***p < 0.001. c) Table indicating top fold-change of average mol% of lipid species differentially modulated by ACM from *APOE3/3* and *APOE4/4* astrocytes, ranked by Tukey's post-hoc for paired comparisons versus NO ACM. Data represent median ± standard deviation. Lipid species were annotated per total acyl carbons and degree of unsaturation.

**Supplemental Figure 1**

**

**

**Supplemental Figure 2**
